# The ribosomal protein S6 kinase alpha-1 (RPS6KA1) induces resistance to venetoclax/azacitidine in acute myeloid leukemia

**DOI:** 10.1038/s41375-023-01951-8

**Published:** 2023-07-06

**Authors:** Katharina Weidenauer, Christina Schmidt, Christian Rohde, Cornelius Pauli, Maximilian F. Blank, Daniel Heid, Alexander Waclawiczek, Anika Corbacioglu, Stefanie Göllner, Michelle Lotze, Lisa Vierbaum, Simon Renders, Jeroen Krijgsveld, Simon Raffel, Tim Sauer, Andreas Trumpp, Caroline Pabst, Carsten Müller-Tidow, Maike Janssen

**Affiliations:** 1 Department of Internal Medicine V, Hematology, Oncology and Rheumatology, University Hospital Heidelberg, Heidelberg, Germany; 2 University of Heidelberg Medical Faculty, Heidelberg, Germany; 3 Molecular Medicine Partnership Unit (MMPU), University of Heidelberg and European Molecular Biology Laboratory (EMBL), Heidelberg, Germany; 4 Division of Mechanisms Regulating Gene Expression, German Cancer Research Center (DKFZ), Heidelberg, Germany; 5 Division of Proteomics of Stem Cells and Cancer, German Cancer Research Center (DKFZ), Heidelberg, Germany; 6 Division of Stem Cells and Cancer, German Cancer Research Center (DKFZ) and DKFZ-ZMBH Alliance, Heidelberg, Germany; 7 Heidelberg Institute for Stem Cell Technology and Experimental Medicine (HI-STEM gGmbH), Heidelberg, Germany

**Keywords:** Acute myeloid leukaemia, Cancer therapeutic resistance, Cancer genomics

## Abstract

Venetoclax/azacitidine combination therapy is effective in acute myeloid leukemia (AML) and tolerable for older, multimorbid patients. Despite promising response rates, many patients do not achieve sustained remission or are upfront refractory. Identification of resistance mechanisms and additional therapeutic targets represent unmet clinical needs. By using a genome-wide CRISPR/Cas9 library screen targeting 18,053 protein- coding genes in a human AML cell line, various genes conferring resistance to combined venetoclax/azacitidine treatment were identified. The ribosomal protein S6 kinase A1 (RPS6KA1) was among the most significantly depleted sgRNA-genes in venetoclax/azacitidine- treated AML cells. Addition of the RPS6KA1 inhibitor BI-D1870 to venetoclax/azacitidine decreased proliferation and colony forming potential compared to venetoclax/azacitidine alone. Furthermore, BI-D1870 was able to completely restore the sensitivity of OCI-AML2 cells with acquired resistance to venetoclax/azacitidine. Analysis of cell surface markers revealed that RPS6KA1 inhibition efficiently targeted monocytic blast subclones as a potential source of relapse upon venetoclax/azacitidine treatment. Taken together, our results suggest RPS6KA1 as mediator of resistance towards venetoclax/azacitidine and additional RPS6KA1 inhibition as strategy to prevent or overcome resistance.

## Introduction

AML is predominantly a disease of the elderly with a median age of 67 years at diagnosis [1]. The underlying genetic aberrations are diverse and change dynamically during disease progression and under therapeutic pressure. Specifically, deregulation of pro-apoptotic and pro-survival cellular signaling leads to a shift towards unregulated proliferation and differentiation. Overexpression of the antiapoptotic protein BCL-2 in AML blasts and leukemic stem cells (LSCs) prohibits caspase- dependent cell death [2] and maintains oxidative phosphorylation on which LSCs depend [3]. Accordingly, high expression levels of BCL-2 are associated with chemoresistance and a worse prognosis of the disease [4]. Intensive induction chemotherapy with cytarabine and daunorubicin enables a five- year overall survival of 35% [5], but especially older patients are not amenable to this therapy regime. This is mainly due to pre-existing comorbidities and reduced general condition associated with increased treatment- related mortality. For these patients, low-intensity therapies with hypomethylating agents (HMAs) or low-dose cytarabine (LDAC) are offered but are less effective with a median overall survival of less than a year [6]. While monotherapy with the BCL-2 inhibitor venetoclax is not effective [7], higher response rates are achieved by the combination of venetoclax with HMAs such as azacitidine or decitabine [2], or LDAC [8]. These combinations achieved approval by the U.S. Food and Drug Administration (FDA) and the European Medicines Agency (EMA) for newly diagnosed AML patients ineligible for intensive chemotherapy [9]. However, the efficacy of combination therapy of venetoclax with HMAs or LDAC is limited by upfront resistance and frequent relapse after initial response [10]. Underlying mechanisms are poorly understood. Venetoclax is a small- molecule inhibitor of BCL-2 which leads to the release of pro-apoptotic members of the BCL-2 family, culminating in mitochondrial outer membrane permeabilization and cytochrome C release [11]. Azacitidine enhances pro-apoptotic signaling by down-regulation of the anti-apoptotic protein MCL-1 [12] and upregulation of apoptosis- promoting proteins such as NOXA and PUMA. Given the inhibition of MCL-1 and BCL-XL, the leukemic cells become more dependent on BCL-2 and therefore are more susceptible to venetoclax treatment [4]. Furthermore, LSCs are targeted by the venetoclax/azacitidine combination through collaborative inhibition of oxidative phosphorylation [13]. One mechanism behind intrinsic or acquired resistance is the overexpression of anti-apoptotic proteins such as MCL-1 or BCL-XL which occurs in AML with FLT3-ITD mutation [10] or loss of *TP53* [14], thereby mitigating the cellular dependence on BCL-2 [15].

In order to identify drug targets to circumvent or overcome resistance towards venetoclax/azacitidine, we performed a genome-wide CRISPR/Cas9 knockout screen in the human AML cell line OCI-AML2. In line with previous studies [16], *PMAIP1* and *BAX* as key regulatory genes in the apoptotic pathway were identified as important mediators of venetoclax/azacitidine response. Among the top hits of genes mediating venetoclax/azacitidine resistance, we found the ribosomal protein S6 kinase A1 (*RPS6KA1*). Pharmacological inhibition of RPS6KA1 increased sensitivity to venetoclax/azacitidine in parental AML cells and could restore sensitivity of venetoclax/azacitidine-resistant AML. Our findings suggest RPS6KA1 as a promising drug target for new combinatorial strategies to circumvent venetoclax/azacitidine resistance in AML.

## Materials and methods

### Cell culture

Human OCI-AML2 (ACC 99), MOLM-13, HL-60, Kasumi-1 and MV-411 cell lines were purchased from German Collection of Microorganisms and Cell Cultures GmbH (DSMZ) and cultured in RPMI-1640 supplemented with 10% (HL60) or 20% fetal bovine serum (FBS). 293 T cells (CRL-3216) were purchased from American Type Culture Collection (ATCC) and cultured in DMEM with 10% FBS. All cell lines were grown at 37 °C with 5% CO_2_ in log phase (1 × 10^5^ to 1 × 10^6^ cells/ml) and split every third day. All cell lines used in this study were tested for mycoplasma and authenticated before the experiments were started. Resistant cell lines were generated by weekly treatment with increasing doses of venetoclax (S8048, Selleckchem, Houston, Texas, USA) and azacitidine (sc-221003, Santa Cruz Biotechnology, Dallas, Texas, USA) for several months (Supplementary Fig. S3A). Treatment was started with 1 nM venetoclax and 5 nM azacitidine and doubled every week. As maintenance dose, resistant OCI-AML2 cells were treated with 300 nM venetoclax and 1 µM azacitidine twice weekly. For validation experiments, BI-D1870 (S2843, Selleckchem, Houston, Texas, USA) and SL1010 (Selleckchem) were used as RPS6KA1 inhibitors and SB216763 (Selleckchem) as GSK3 inhibitor.

Primary human bone marrow samples were obtained from AML patients who provided informed consent. Biobank procedures are approved by the “Ethikkomission Heidelberg”. Informed consent was obtained from all patients. Primary cells were density gradient-isolated and cultured as described in [17]. Patients` information is given in Supplementary Table S1.

### CRISPR/Cas9 library screen

A genome-wide CRISPR/Cas9 knockout screen was conducted in the leukemic cell line OCI-AML2 in the presence or absence of venetoclax and azacitidine. The one-component Toronto KnockOut library v3 (LCV2::TKOv3, 90294, Addgene, Watertown, Massachusetts, USA) harboring 70 948 single-guide RNAs (sgRNA) targeting 18 053 protein- coding genes with 4 gRNAs per gene as well as 142 control non-targeting guides against EGFP, LacZ and luciferase was used. The screen was performed according to the protocol of the Moffat Lab from the university of Toronto with small modifications [18]. In brief, the initial library was amplified in Endura electrocompetent cells while packaging plasmid psPAX2 (12260, Addgene, Watertown, Massachusetts, USA) and envelope plasmid pMD2.G (12259, Addgene, Watertown, Massachusetts, USA) were amplified in E. coli stable 3 cells. Subsequently, 293 T cells were transfected with the TKOv3, psPAX2 and pMD2.G using TurboFect (R0532, Thermo Fisher Scientific, Waltham, Massachusetts, USA). In total, 18 h after transfection, the medium was replaced with a serum-free high-BSA growth medium. After another 24 and 48 h, the virus- containing supernatant was harvested and concentrated via ultracentrifugation. The human OCI-AML2 cell line was infected with the TKOv3 library via lentiviral transduction with a library coverage of at least 200-fold and with a multiplicity of infection of 0.3. Three days after 72 h puromycin selection, the input control samples (time zero, T0) were harvested in triplicates. The remaining effectively transduced cells were then treated with venetoclax/azacitidine at the IC_60_ to IC_70_ concentration or with DMSO as a control to compensate for cytotoxic effects of the solvent. 14 days after start of treatment, samples were taken with three technical replicates. For each sample, genomic DNA was isolated and a two-step polymerase chain reaction (PCR) was performed, maintaining a 200-fold coverage of the TKOv3 library. Primer sequences are listed in the supplements (Supplementary Table S2). In the first PCR (25 cycles), guide-RNA regions in the genome were enriched and then amplified in the second PCR (10 cycles) using unique combinations of dual indices and stagger sequences as well as Illumina sequencing adapters that allow multiplex sequencing. The PCR products were purified with Agencourt AMPure XP beads (A63881, Beckman Coulter, Brea, California, USA) and submitted to high-throughput sequencing on an Illumina NextSeq500. The sequencing data was then demultiplexed and the MaGeCK algorithm [19] was applied to identify differences in sgRNA abundance between treatment and control group at different time points and to generate a sgRNA-ranking based on p-values (Supplementary Table S3).

### Gene set enrichment analysis

The dataset was ranked by log fold change (LFC) values for each gene and then subjected to gene set enrichment analysis (GSEA) using the R package fgsea version 1.14.0 [20]. The gene sets were further specified using the R package reactome.db version 1.70.0 [21]. Default settings with a minimum size of gene sets of 15, a maximum size of gene sets of 500, and the number of permutations set to 10 000 were used for the analysis.

### Western blot analysis

Cell pellets were lysed in RIPA-buffer supplemented with protease and phosphatase inhibitor. The protein concentration was determined with a Pierce BCA Protein Assay Kit (23227, Thermo Fisher Scientific, Waltham, Massachusetts, USA). Equal amounts of protein were used for gel electrophoresis and blotted on a nitrocellulose membrane. Membranes were incubated at 4 °C overnight with anti-B-ACTIN (1:5 000, A5441, Sigma Aldrich), anti-GSK-3 α/ß (1:200, sc-7291, Santa Cruz Biotechnology, Dallas, Texas, USA), anti-MCL-1 (1:4 000, ab32087, abcam), anit-pGSK-3α/ß Ser21/9 (1:1 000, 8566, Cell Signaling Technology), anti-RPS6KA1 (ab32114, Abcam, Cambridge, UK; 1:1 000) and secondary antibodies against mouse or rabbit immunoglobulin (1:4 000, P044701-2, P044801-2, Dako/Agilent, Santa Clara, California, USA). Proteins were visualized on an Amersham imager 600 (Cytiva, Chalfont St Giles, UK) using ECL reagent. All images have been cropped for improved clarity and conciseness. Densitometry analysis was performed using ImageJ.

### Drug sensitivity assays and cell viability assessment

Cells were plated in 96-well plates at a density of 1 × 10^4^ cells per well and exposed to increasing drug concentrations. After 48 or 72 h, cell viability was read out with a CellTiter 96 AQueous One Solution Cell Proliferation Assay (G3582, Promega, Fitchburg, Massachusetts, USA) on a plate reader (Tecan, Männedorf, Switzerland). Absolute absorbance values were normalized to DMSO control treatment and IC_50_ values were computed in GraphPad Prism version 9.2.0 with nonlinear fit of [Inhibitor] versus response (three parameters). For the IC_50_ determination of venetoclax/azacitidine, a compound was mixed at a fixed ratio of 1:4 before adding the combination in a dilution series to the AML cells. To identify synergistic effects of drug combinations, a synergy score was computed using the Bliss Independence Model [22]. According to this algorithm, synergism is affirmed if the observed response to combined drug treatment is larger than the predicted response assumed as the sum of single drug treatment [22].

Primary cells were seeded with a density of 5 × 10^4 cells per well. Viability was determined by staining cells with trypan blue after 48 h of cultivation and counting viable and dead cells.

### Proliferation assay

The proliferation of leukemia cells was quantified by serial cell counting every 24 h for 5 days. Cells were seeded with a density of 0.25 ×10^6^ per ml, plated in different treatment groups with three technical replicates and grown in log phase (1 × 10^5^ to 1 × 10^6^ cells/ml) during the assay.

### Apoptosis assay and intracellular staining

Apoptosis and necrosis were measured by staining 0.5 × 10^5^ cells per sample with Annexin V and propidium iodide (556547, BD biosciences, Franklin Lakes, New Jersey, USA) followed by flow cytometric analysis with a FACS Celesta cell analyzer (BD biosciences, Franklin Lakes, New Jersey, USA). For primary samples, cells were seeded at a density of 5 × 10^4^ cells per well in a 96-well plate and treated with the respective drugs for 24 h. Experiments were accomplished with three technical replicates and each experiment was performed at least twice.

Intracellular staining of anti-apoptotic proteins was performed as in [23].

### Colony-forming unit (CFU) assay

In order to quantify the colony formation capacity of cells under different treatment conditions, 300 cells (cell line samples) or 4000 cells (primary samples) were seeded in 600 μl methylcellulose (04230 or 04234, Stemcell technologies, Vancouver, Canada) supplemented with 10% FBS, penicillin streptomycin and the indicated drugs into twelve-well plates. Colonies with at least 50 cells were quantified after eight- and ten- day incubation, respectively, at 37 °C with 5% CO_2_. All experiments were performed in technical triplicates and each experiment was performed at least three times.

### Cell surface marker analysis

To further characterize different subpopulations by their lineage or differentiation status upon CFU assays, cells were isolated by resuspending the methylcellulose in media and washing. Cells were then stained with a broad panel of myeloid markers: anti-GPR56 (358204, BioLegend, San Diego, California, USA), anti-CD11b (301344, BioLegend, San Diego, California, USA), anti-CD64 (305014, BioLegend, San Diego, California, USA), anti-CD14 (612902, BD, Franklin Lakes, New Jersey, USA), anti-CD33 (25-0338-42, LIFE Technologies, Carlsbad, California, USA) and anti-CD117 (313238, BioLegend, San Diego, California, USA). Stainings were analyzed by flow cytometry.

### Proteomics

For mass spectrometric analyses of parental and resistant cell lines, cells were plated at a density of 5 × 10^5^ cells per ml. After 48 h, 1.5 × 10^7^ cells were pelleted and lysed, and thereafter the cell lysates were sonicated for 15 cycles. After centrifugation, supernatants containing proteins were reduced by addition of dithiothreitol (DTT) and alkylated by addition of chloroacetamide (CAA). Trypsin and a recombinant serine protease were added for digestion overnight. Then, the samples were acidified to stop the proteolytic reaction. After centrifugation, supernatants containing peptides were analyzed on a Tri‐Hybrid Orbitrap Fusion mass spectrometer (Thermo Fisher Scientific, Waltham, Massachusetts, USA). All experiments were performed with three biological replicates. The raw data were processed using MaxQuant (22). To filter the identified proteins, proteins were aligned with a database containing protein sequences of contaminants, enzyme specificity was determined with a maximum of two missed cleavages allowed, and fixed as well as variable modification were selected. The global false discovery rate for both protein and peptides was set at 1%. The normalized label-free quantification (LFQ) values of the remaining proteins with a minimum of one unique peptide were used for further comparative analyses. A *p* value ranking of proteins was submitted to GSEA using the R package fgsea version 1.14.0 [20] and gene sets were defined by the R package ReactomePA version 1.24.0 [24].

### Software and statistics

R version 4.0.4 [25] and Rstudio version 1.4.1106 [26] were used for further analysis of the sequencing data as well as GSEA. Statistical evaluation and graphical reports were established with Graphpad Prism version 9.2.0 (GraphPad Software, San Diego, California, USA) and a student’s *t* test was calculated to evaluate statistical significance of the results. Adobe Illustrator version 25.4.1 and Biorender (BioRender.com) were used for graphics.

## Results

### A genome-wide CRISPR/Cas9 library screen identified RPS6KA1 as potential mediator of venetoclax/azacitidine resistance in AML in vitro

**Fig. 1:**
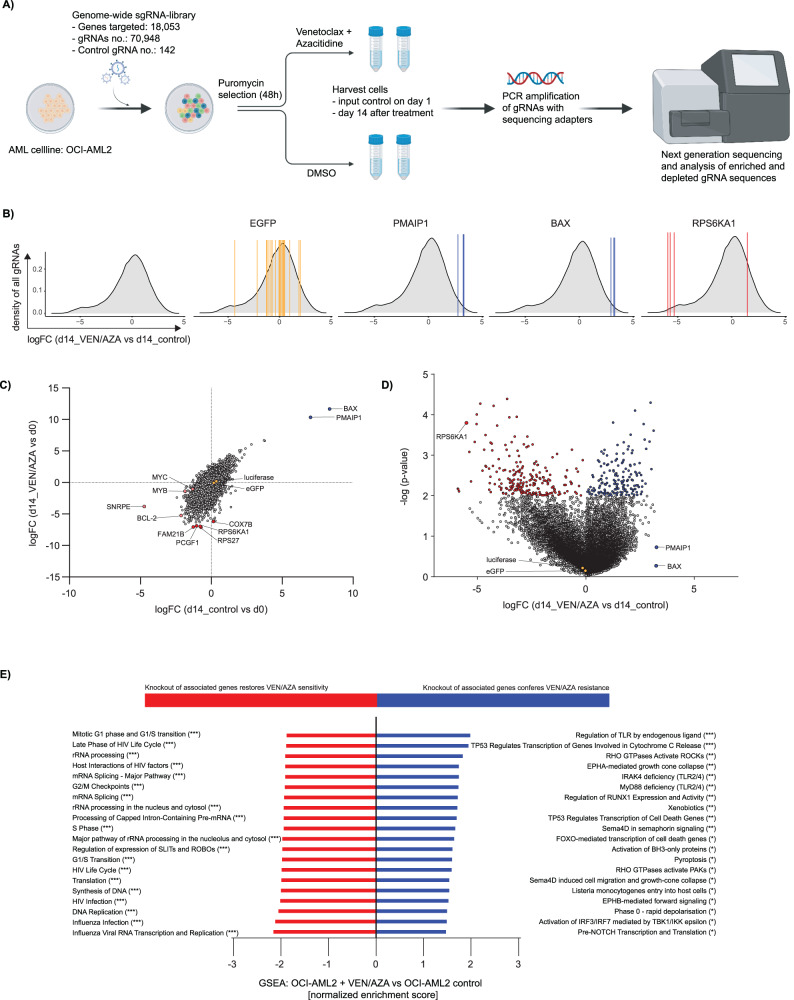
A genome-wide CRISPR/Cas9 library screen identified genes associated with resistance and sensitivity towards venetoclax/azacitidine in AML cells. **A** Overview of the experimental design and workflow of the CRISPR/Cas9 library screen: OCI-AML2 cells were infected with the genome-wide Toronto KnockOut sgRNA library v3 and treated either with 20 nM venetoclax and 50 nM azacitidine or DMSO. Samples were taken at day 1 and 14 after start of treatment. Genomic DNA was PCR- amplified and the relative abundance of sgRNAs was determined by next generation sequencing. This figure was created with BioRender.com. **B** Density plots showing the average distribution of log-fold change values for all sgRNAs (left) between treatment and control group on day 14, and single sgRNAs (middle to right) demonstrating the concordance among different sgRNAs targeting a given gene. The single sgRNAs were highlighted for eGFP as non-targeting control, *PMAIP1* and *BAX* as positively enriched sgRNA-genes and *RPS6KA1* as negatively enriched sgRNA-gene. **C** Log-fold change values of all sgRNA-genes from day 14 to day 0. The comparison of sgRNA abundance in venetoclax/azacitidine vs. DMSO treated samples revealed genes associated with resistance or sensitivity towards venetoclax/azacitidine treatment. Blue and red color encodes positive and negative enrichment, respectively. Orange color denotes non-targeting sgRNA-genes. **D** Volcano plot showing log-fold change values and significance of all sgRNA-genes from day 14 in venetoclax/azacitidine vs. DMSO treated cells. *RPS6KA1* is among the strongest depleted sgRNA-genes in venetoclax/azacitidine- treated cells while *PMAIP1* and *BAX* are positively enriched. Non-targeting sgRNAs for luciferase and eGFP are not altered by drug stimulation. **E** Gene set enrichment analysis of sgRNA-genes ranked by their LFC values. The top 20 significantly upregulated (filtered for a gene set size larger than 100 genes) or downregulated gene sets in venetoclax/azacitidine- treated OCI-AML2 cells vs. untreated control cells (T14) are shown. *p < .05, **p .01, ***p .001. Statistical significance was determined by a two-sided unpaired Student`s *t* test. The variance was similar between the groups.

To identify genes associated with treatment response or resistance, we transduced the human AML cell line OCI-AML2 with the one-component Toronto KnockOut library v3 harboring 70,948 single-guide RNAs (sgRNA) targeting 18 053 protein- coding genes as well as 142 control non-targeting guides. The effectively transduced cells were selected by puromycin treatment and then cultured in the presence of venetoclax/azacitidine at their IC_60_ to IC_70_ concentration or DMSO as a control for 14 days. Genomic DNA was isolated, PCR amplified (Supplementary Table S2), and submitted to deep sequencing of integrated gDNAs (Supplementary Fig. S1A). For analysis of sgRNA distribution before and after 14 days of venetoclax/azacitidine treatment, the MaGeCK algorithm—a Model-based Analysis of Genome-wide CRISPR-Cas9 Knockout—was applied (Fig. 1A) [19].

The log-fold change values (LFC) of sgRNAs targeting one selected gene were compared to the average distribution of LFC values of all sgRNAs between treatment and control group on day 14 (Fig. 1B). Only genes with high concordance among sgRNAs targeting a given gene were included. sgRNAs targeting genes that mediate resistance or enhance sensitivity to venetoclax/azacitidine treatment are proposed to be negatively or positively selected, respectively, upon drug stimulation compared to the DMSO treated cells (Supplementary Fig. S1B). Knockout of common essential genes such as *MYC* and *MYB* but also *BCL-2*, the target of venetoclax, led to depletion of cells with and without venetoclax/azacitidine stimulation while abundance of sgRNAs targeting control genes luciferase and eGFP was not altered (Fig. 1C). AML cells with depletion of *BAX* and *PMAIP1* (*NOXA*) as key regulator genes in the apoptosis cascade and thus important mediators of venetoclax response showed the strongest proliferation with and without drug stimulation (Fig. 1C, Supplementary Fig. S1C). Accordingly, sgRNAs targeting *BAX* and *PMAIP1* showed the highest positive LFC values. These results confirmed the validity of our screen (Supplementary Table S4 and S5). Since the identification of genes mediating resistance to venetoclax and azacitidine was the main aim of the screening approach, we were particularly interested in depleted sgRNAs in venetoclax/azacitidine- treated cells 14 days after treatment initiation (Fig. 1D, Supplementary Fig. S1D, Supplementary Table S6). A gene ranking of statistically significantly depleted sgRNAs (*p* < 0.05), based on their LFC ≤ -2 compared to DMSO treated cells and with a high concordance of at least 75%, identified 504 genes. sgRNAs targeting the ribosomal protein S6 kinase A1 (RPS6KA1) were identified consistently among the strongest negatively enriched hits. In a ranking by LFC values and MaGeCK score RPS6KA1- sgRNA was on the fourth and seventh rank, respectively, with a LFC of –5.5056 and reached statistical significance with a *p* value of 0.0002. The concordance among sgRNAs targeting this gene with three out of four sgRNAs showing the same effect is indicative for a high validity of the observed effect.

We compared the results from our screen with three previously conducted CRISPR/Cas 9 screens investigating resistance against venetoclax mono treatment. TP53, BAX and PMAIP1 were constantly identified among the top enriched genes (high negative rank) in the different venetoclax mono CRISPR/Cas9 screens as well as in the venetoclax/azacitidine CRISPR/Cas9 screen presented in this study [11, 16, 27] (Supplementary Fig. S2A). Previously, CLPB [11] and DAP3 [27] were further validated as genes mediating resistance to venetoclax therapy, while TP53 was identified as mediator of venetoclax response [16]. When comparing the median MaGeCK rank for negative enrichment of sgRNAs targeting TP53, BAX, PMAIP1 and RPS6KA1 in the different screens, the three previously named genes reached a similar ranking whereas negative enrichment of RPS6KA1 was mainly found in the venetoclax/azacitidine screen (Supplementary Fig. S2B). These data indicate that RPS6KA1 could specifically mediate venetoclax/azacitidine resistance but not venetoclax mono resistance.

Taken together, RPS6KA1 was identified as a promising candidate gene conferring resistance to venetoclax/azacitidine combination therapy and was chosen for further validation experiments.

### Venetoclax/azacitidine resistance is associated with genes involved in translation, mRNA splicing and MAPK signaling

To identify functional pathways involved in venetoclax/azacitidine resistance, a list of sgRNA-genes ranked by LFC values in venetoclax/azacitidine- vs. DMSO- treated samples was evaluated by gene set enrichment analysis (GSEA). The results implied that depletion of sgRNAs targeting translation-associated genes might have a functional relevance for proliferation under venetoclax/azacitidine treatment (Fig. 1E). Furthermore, knockout of genes associated with DNA replication, cell cycle progression, and mRNA splicing as well as rRNA processing might also confer susceptibility to venetoclax/azacitidine treatment (Fig. 1E). In contrast, cells harboring a knockout of genes involved in transcriptional regulation by *TP53* signaling or activation of BH3-only proteins had a proliferative advantage (Fig. 1E). GSEA of the CRISPR screen dataset also revealed that sgRNAs targeting genes involved in MAPK signaling were depleted upon venetoclax/azacitidine stimulation (Supplementary Fig. S2C). *RPS6KA1* encodes for an 82.7 kDa serine/threonine kinase located in the nucleus and cytoplasm which is associated with the MAPK/ERK (MAPK1/ERK2, MAPK3/ERK1) complex. In conclusion, the association of venetoclax/azacitidine resistance with MAPK signaling pointed to a possible functional relevance of RPS6KA1.

### Inhibition of RPS6KA1 increases the efficacy of venetoclax/azacitidine in AML

**Fig. 2:**
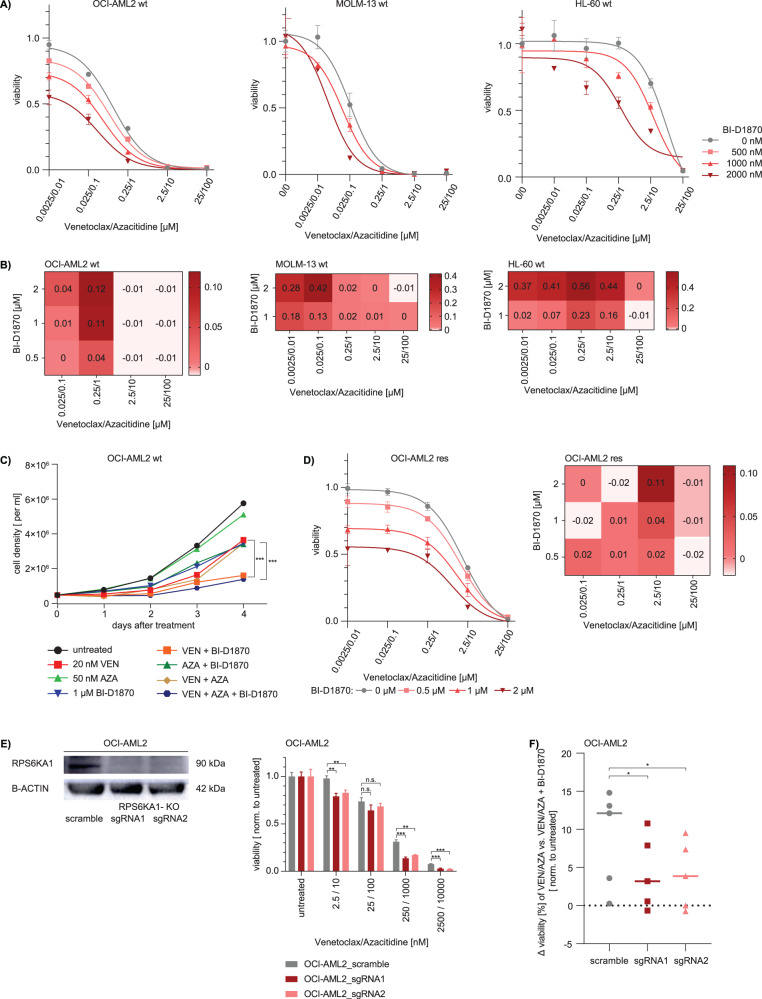
Pharmacological inhibition of RPS6KA1 as well as RPSKA1 knockout decreases cell proliferation in different AML cell lines and RPS6KA1 inhibition resensitizes resistant AML cells to venetoclax/azacitidine treatment. **A** Viability assay and IC_50_ measurements. Parental (wt) OCI-AML2, MOLM-13, HL-60 cells were treated with a premixed combination of venetoclax/azacitidine in serial dilutions and different concentrations of the RPS6KA1 inhibitor BI-D1870. Cell viability was assessed upon drug exposure for 72 h and normalized to DMSO control. Depicted are means ± SD from three technical replicates. Data are representative for 3 independent experiments. **B** Synergism was calculated for different drug combinations of venetoclax/azacitidine with BI-D1870 in parental (wt) OCI-AML2, MOLM-13, HL-60 cells. The synergy score was computed according to the Bliss Independence Model and depicted as a heatmap. Positive and negative Bliss Scores are indicative for synergism and antagonism, respectively, while neutral Bliss Scores are often confounded by strong single drug effects. **C** Proliferation assay. Parental (wt) OCI-AML2 cells were seeded with a density of 0.5 × 10^6^ per ml and treated either with venetoclax, azacitidine, BI-D1870 or different combinations of the three drugs. Cell number was quantified by serial cell counting over 4 days. Depicted are means from three technical replicates. Data are representative for 2 independent experiments. *p < 0.05, **p < 0.01, ***p < 0.001. Statistical significance was determined by a two-sided unpaired Student`s *t* test. The variance was similar between the groups. **D** Viability assay, IC_50_ measurements and synergy calculation. Resistant (res) OCI-AML2 cells were treated with a premixed combination of venetoclax/azacitidine in serial dilutions and different concentrations of the RPS6KA1 inhibitor BI-D1870. Cell viability was assessed upon drug exposure for 48 h and normalized to DMSO control. Depicted are means ± SD from three technical replicates. The synergy score was computed according to the Bliss Independence Model and depicted as a heatmap. Positive and negative Bliss Scores are indicative for synergism and antagonism, respectively, while neutral Bliss Scores are often confounded by strong single drug effects. Data are representative for three independent experiments. **E** RPS6KA1 knockout was established in OCI-AML2 cells and validated via western blot. Those cells were treated with a premixed combination of venetoclax/azacitidine in serial dilutions. Cell viability was assessed upon drug exposure for 72 h and normalized to untreated control. Depicted are means ± SD from three technical replicates. Data are representative for three independent experiments. **F** The difference of viability in cells treated with different doses of venetoclax/azacitidine attained upon the addition of RPS6KA1 inhibition to the treatment was calculated and compared for OCI-AML2 scramble cells versus OCI-AML2 cells with RPS6KA1 knockout. Data was calculated from means from three technical replicates.

To further characterize the role of RPS6KA1 as a mediator of proliferation and therapy resistance in AML, we performed functional assays using pharmacological inhibition of RPS6KA1 with the RSK inhibitor BI-D1870. Functional assays were performed in various AML cell lines. A measure of single drug efficacy is the half maximal inhibitory concentration (IC_50_). To establish a measure of efficacy also for the venetoclax/azacitidine combination, both drugs were mixed in a 1:4 ratio. Next, several AML cell lines were treated with the pre-mixed compound in the presence or absence of the RSK inhibitor BI-D1870. Upon drug exposure for 72 h, a dose-dependent reduction of venetoclax/azacitidine IC_50_ with increasing concentrations of BI-D1870 was identified in OCI-AML2, MOLM-13 and HL60 cells (Fig. 2A, Supplementary Fig. S2D). The combination of all three drugs showed synergistic effects indicated by a positive Bliss Score (21) of up to 0.56 (Fig. 2B). The strongest synergistic effects were observed in MOLM-13 and HL-60 cells with 2 µM BI-D1870, 25 / 250 nM venetoclax and 100 / 1000 nM azacitidine. Neutral Bliss Score at higher concentrations of venetoclax/azacitidine resulted from a strong effect of each drug individually, preventing synergy of the combination. Similar results were observed in further AML cell lines, when MV4-11 and Kasumi-1 cells were tested to validate these results (Supplementary Fig. S2D, S2E, S2F).

To further assess BI-D1870 effects on proliferation, OCI-AML2 cells were serially counted every 24 h for several days. While untreated cells and those treated with 50 nM azacitidine had the highest proliferation rate, the addition of 1 µM BI-D1870 to 20 nM venetoclax as well as to the venetoclax/azacitidine combination significantly reduced proliferation (Fig. 2C). Also, inhibition of RPS6KA1 re-sensitized venetoclax/azacitidine- resistant OCI-AML2 cells and knockout of RPS6KA1 affected viability of cells (Fig. 2D–F).

**Fig. 3:**
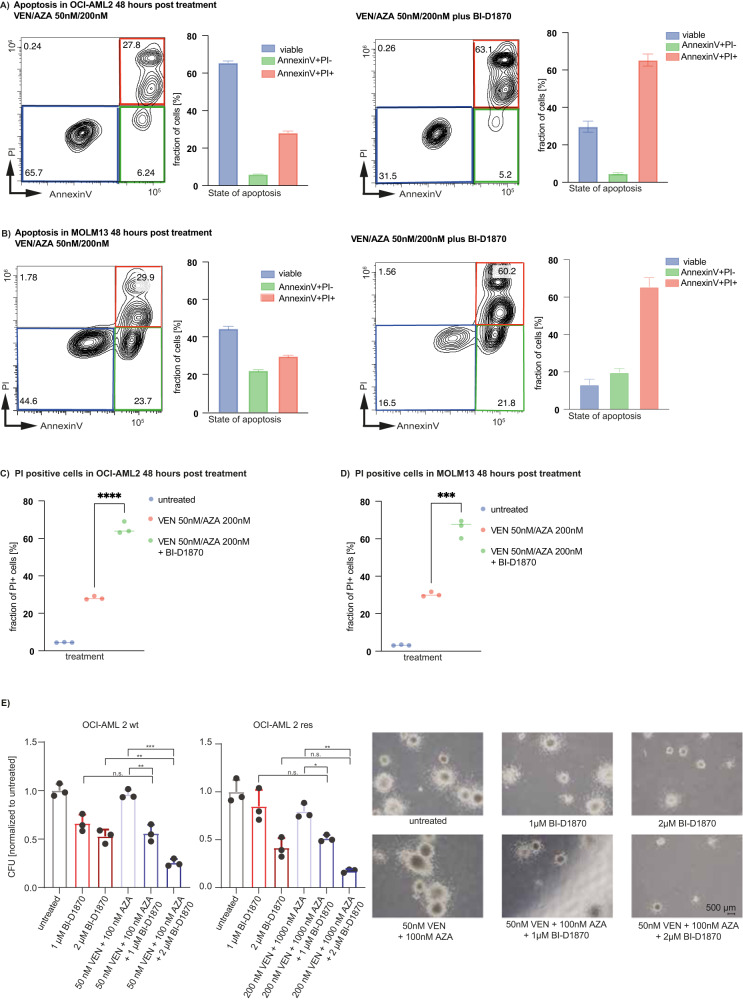
Inhibition of RPS6KA1 increases apoptosis and diminishes colony formation capacity when combined with venetoclax/azacitidine. Apoptosis assay. Parental (wt) OCI-AML2 (**A**) and MOLM-13 (**B**) cells were treated with venetoclax/azacitidine with or without BI-D1870 for 48 h. Cells were stained with Annexin V-pacific blue as an apoptosis marker and propidium iodide as a necrosis marker and analyzed by flow cytometry. The blue boxes and bars indicate the fraction of viable cells. The green boxes and bars indicate the fraction of cells undergoing apoptosis. The red boxes and bars indicate the fraction of cells undergoing necrosis. FACS plots are representative for one technical replicate out of three and two independent experiments. **C** Comparison of the fraction of PI positive OCI-AML2 cells in untreated cells (median: 4.44), cells treated with either venetoclax/azacitidine (median: 27.8) or venetoclax/azacitidine with BI-D 1870 (median: 63.9), ****p < 0.0001. Statistical significance was determined by a two-sided unpaired Student`s *t* test. The variance was similar between the groups. **D** Comparison of the fraction of PI positive MOLM-13 cells in untreated cells (median: 3.12), cells treated with either venetoclax/azacitidine (median: 29.9) or venetoclax/azacitidine with BI-D 1870 (median: 67), ***p 0.0003. Statistical significance was determined by a two-sided unpaired Student`s *t* test. The variance was similar between the groups. **E** Colony forming unit (CFU) assay with parental (wt) and resistant (res) OCI-AML2 cells. 300 cells were seeded in methylcellulose and treated with venetoclax/azacitidine, BI-D1870 or the combination of all three drugs with different venetoclax/azacitidine concentrations used in wildtype (wt) and resistant (res) OCI-AML2 cells. Colonies with at least 50 cells were quantified 8 days after seeding and normalized to untreated samples. Depicted are means ± SD from three technical replicates. Data are representative for three independent experiments. Representative images are shown from colonies of parental (wt) OCI-AML2 cells. *p < 0.05, **p 0.01, ***p 0.001. Statistical significance was determined by a two-sided unpaired Student`s *t* test. The variance was similar between the groups.

As RPS6KA1 has been reported to mediate cell survival by phosphorylating and thus inhibiting the pro-apoptotic proteins BAD and DAPK1 [28, 29], we questioned whether BI-D1870 treatment might induce apoptotic cell death of AML cells. The reduced viability was associated with enhanced apoptosis. Apoptosis and necrosis were measured upon drug treatment with different doses of venetoclax or azacitidine with and without 2 µM BI-D1870 or the triple combination for 48 and 72 h (Fig. 3A–D, Supplementary Fig. S3A–F). Venetoclax/azacitidine treatment induced apoptosis in OCI-AML2 and MOLM-13 cells (Fig. 3A, B), which was significantly enhanced by the venetoclax/azacitidine and BI-D1870 triple therapy (Fig. 3C, D). Of note, venetoclax combined with BI-D1870 was almost as efficient as the triple combination which was reflected by the amount of apoptosis (Supplementary Figs. S3A-B and S3E). The single effect of azacitidine was not as strong as observed for venetoclax (Supplementary Figs. S3C-D and S3F). Next, we examined the effect of BI-D1870 on colony formation capacity. We analyzed venetoclax/azacitidine treatment and the triple combination including BI-D1870 in a semi-solid medium with cytokine supplementation. Parental OCI-AML2 cells were plated in methylcellulose, treated with 1 μM and 2 μM BI-D1870, 50 nM/100 nM venetoclax/azacitidine or the triple combination, and colonies were counted after eight days. Both proliferation and clonogenic potential were reduced under venetoclax/azacitidine/BI-D1870 triple therapy. While 50 nM/100 nM venetoclax/azacitidine did not significantly reduce clonogenic potential in parental OCI-AML2 cells, treatment with 2 µM BI-D1870 decreased clonogenic potential to 53% compared to untreated samples (Fig. 3E). The triple combination of venetoclax, azacitidine and BI-D1870 further reduced clonogenic potential to 25% compared to untreated control (Fig. 3E). Not only the number of colonies but also their size was reduced under triple therapy compared to cells treated with venetoclax/azacitidine (Fig. 3E). In conclusion, addition of BI-D1870 to venetoclax/azacitidine could further impede proliferation and colony formation of parental AML cells.

In order to investigate the off-target effects of BI-D1870, we tested the effects of SL-1010, another RPS6KA1 inhibitor, on the viability of OCI-AML2 cells. When combined with venetoclax/azacitidine, viability was decreased to a similar degree by SL-1010 as with BI-D1870 although higher doses had to be applied (Supplementary Fig. S4A).

### Inhibition of RPS6KA1 overcomes acquired venetoclax/azacitidine resistance

To investigate the role of RPS6KA1 in acquired venetoclax/azacitidine resistance, we analyzed OCI-AML2 cells that were rendered resistant by continuous exposure to increasing doses of venetoclax/azacitidine. Resistant OCI-AML2 cells tolerated concentrations of more than 200 nM venetoclax and 1 µM azacitidine (Supplementary Fig. S4B). The combined IC50 for venetoclax/azacitidine was 42.44 in parental OCI-AML2 cells compared to 776.9 in resistant OCI-AML 2 cells (Supplementary Fig. S2D). Addition of the RSK inhibitor BI-D1870 restored sensitivity to venetoclax/azacitidine in resistant cells (Fig. 2D, Supplementary Fig. S2D). However, in resistant OCI-AML2 cells, higher concentrations of venetoclax and azacitidine were needed to observe a synergism with BI-D1870 (Fig. 2D).

To determine the specificity of RPS6KA1 inhibition, we established a CRISPR/Cas9- mediated RPS6KA1 knockout in OCI-AML2 cells (Fig. 2E). In OCI-AML2 cells, treatment with venetoclax and azacitidine was significantly more effective in cells harboring a RPS6KA1 knockout compared to scrambled control cells (Fig. 2E). Additional treatment with BI-D1870 had significantly less effects in RPS6KA1 knockout cells compared to wildtype, precluding off-target effects of this inhibitor (Fig. 2F).

Further, addition of BI-D1870 to venetoclax/azacitidine significantly reduced clonogenic potential in resistant OCI-AML2 cells compared to venetoclax/azacitidine alone (Fig. 3E).

### Inhibition of RPS6KA1 increases GSK3-mediated phosphorylation of MCL-1 at serine 159 with consecutive degradation of MCL-1

**Fig. 4:**
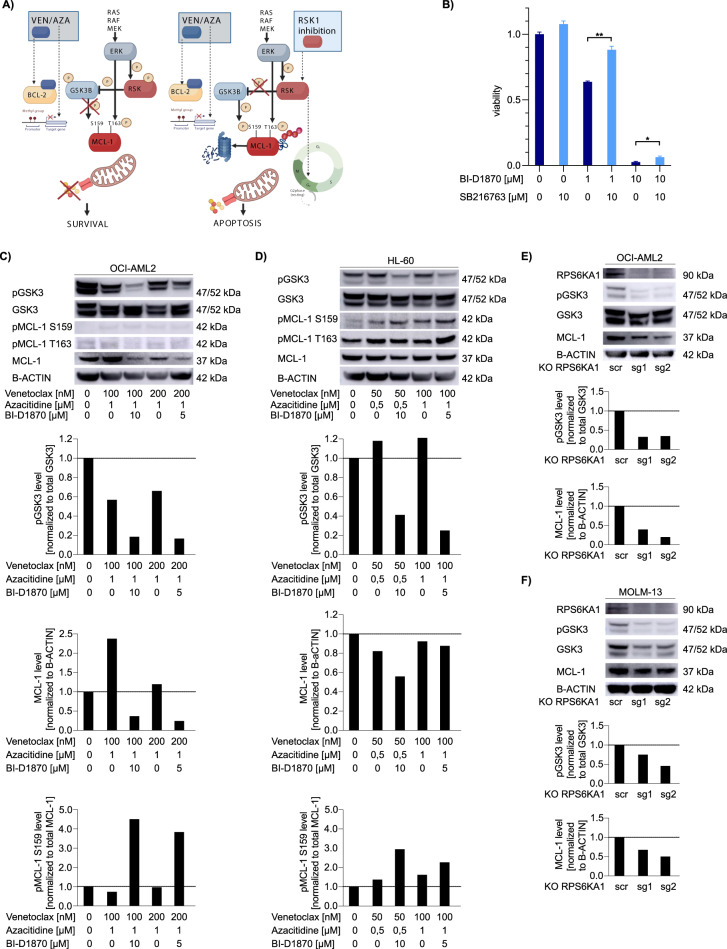
RPS6KA1 inhibition increases the activity of GSK3, increases phosphorylation of MCL-1 at serine 159 and reduces total MCL-1 levels. **A** Schematic overview on the proposed mechanism of action of combining venetoclax/azacitidine with inhibition of RPS6KA1. **B** OCI-AML2 cells were treated with the RPS6KA1 inhibitor BI-D1870 and/or the GSK3 inhibitor SB216763. Depicted are means ± SD from three technical replicates. Data are representative for three independent experiments. *p < .05, **p .01, ***p .001. Statistical significance was determined by a two-sided unpaired Student`s *t* test. The variance was similar between the groups. OCI-AML2 (**C**) and HL-60 (**D**) cells were treated with given dose of venetoclax, azacitidine with/without BI-D1870 for 16 h. Protein expression of pGSK3, GSK3, pMCL-1 S159, pMCL-1 T163 and MCL-1 were analyzed by western blotting. B-ACTIN levels are given as loading control. Quantification was performed using ImageJ. Data are representative for two independent experiments. OCI-AML2 (**E**) and MOLM-13 (**F**) cells were transduced with plasmids for either scramble control or knockout of RPS6KA1 (two different sgRNAs). Protein expression of pGSK3, GSK3 and MCL-1 were analyzed by western blotting. B-ACTIN levels are given as loading control. Quantification was performed using ImageJ. Data are representative for two independent experiments.

In order to investigate the molecular mechanisms responsible, the antiproliferative/pro-apoptotic effects of RPS6KA1 inhibition, we treated OCI-AML2 cells with the RPS6KA1 inhibitor BI-D1870 with or without an GSK3 inhibitor for 72 h. RPS6KA1 regulates different substrates involved in cell survival, growth, and proliferation such as glycogen synthase kinase-3 (GSK3) [30–32]. We suspected that RPS6KA1 mediates the effect of ERK on GSK3 resulting in increased phosphorylation and inhibition of the kinase (Fig. 4A). Indeed, addition of the GSK3 inhibitor diminished anti-proliferative effects of BI-D1870 in OCI-AML2 cells (Fig. 4B) suggesting that a functional consequence of RPS6KA1 inhibition is increased activity of GSK3. Next, we treated OCI-AML2 and HL-60 cells with different doses of venetoclax/azacitidine with or without addition of BI-D1870 for 16 h and analyzed pGSK3 levels by western blot. In both cell lines, we found reduced phosphorylation of GSK3 (Fig. 4C, D) indicating increased activity of GSK3. GSK3 has been shown to phosphorylate MCL-1 at serine 159 and thereby increase proteasomal degradation of the anti-apoptotic protein [14, 33–35]. Upon treatment with venetoclax/azacitidine plus BI-D1870 pMCL-1 serine 159 was increased when compared to total MCL-1 levels (Fig. 4C, D). Also, total MCL-1 levels decreased when cells were treated with venetoclax/azacitidine and BI-D1870 (Fig. 4C, D). The phosphorylation of MCL-1 at threonine 163 that has a stabilizing effect and is mediated by ERK [14, 32, 34] was not affected by treatment with BI-D1870 (Supplementary Fig. S4C-D). For further validation of our findings, we generated OCI-AML2 and MOLM13 clones with a knockout of RPS6KA1 using two different sgRNAs (Figs. 2E, 4F). In both cases, the knockout led to a decreased phosphorylation of GSK3 and to diminished levels of MCL-1 (Fig. 4E, F). Thus, we suggest that inhibition of RPS6KA1 leads to increased activity of GSK3 resulting in a higher degree of phosphorylation of MCL-1 at serine 159. Consequently, MCL-1 levels decrease due to proteasomal degradation (Fig. 4A). As upregulation of MCL-1 is known to be the most important mediator of resistance towards venetoclax [36, 37], RSK-inhibitor induced loss of MCL-1 is crucial for increasing sensitivity to venetoclax/azacitidine.

**Fig. 5:**
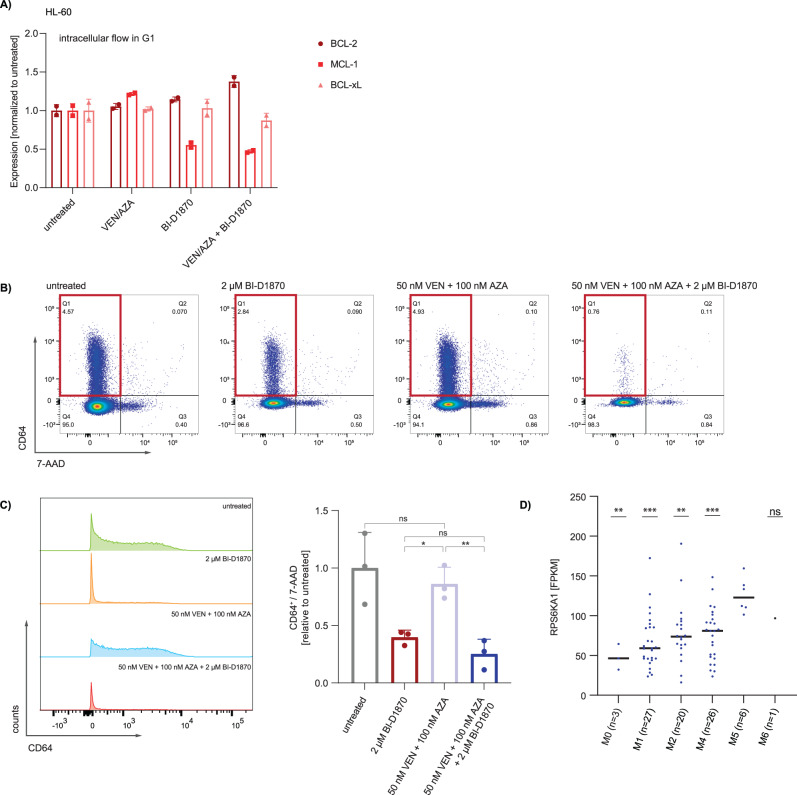
Pharmacological inhibition of RPS6KA1 targets monocytic subpopulations which are resistant to venetoclax/azacitidine treatment. HL-60 (**A**) cells were treated with venetoclax (100 nM in OCI-AML2; 50 nM in HL-60) and azacitidine (1000 nM in OCI-AML2; 500 nM in HL-60) with or without 10 µM BI-D1870 for 16 h. Levels of BCL-2, BCL-XL and MCL-1 were determined by intracellular staining and analysis via flow cytometry. Analysis was carried out for cells in G1 state. Data is shown as mean ± s.d. from three technical replicates. Data are representative for two independent experiments. **B** Flow cytometric analysis of cell surface markers. Parental (wt) OCI-AML2 cells were isolated after CFU assays under different treatment conditions, stained with a panel of myeloid differentiation markers (CD11b, CD64, CD117, GPR56 and CD34) and analyzed by flow cytometry. FACS plots are representative for one technical replicate out of three showing expression levels of the monocytic cell surface marker CD64 and the vital marker 7-AAD. **C** Fraction of CD64^+^/7-AAD^-^ subpopulation in parental (wt) OCI-AML2 cells isolated from CFU assays upon treatment with venetoclax/azacitidine, BI-D1870 or the combination of all three drugs. Depicted are means ± SD from three technical replicates. *p < .05, **p .01, ***p .001. Statistical significance was determined by a two-sided unpaired Student`s *t* test. The variance was similar between the groups. **D** mRNA expression levels of *RPS6KA1* in AML patient samples subdivided by the corresponding FAB category. *RPS6KA1* is expressed at higher levels in monocytic AML subtype M5 compared to AML from other FAB categories. Expression data are obtained from Zhou et al. [50]. All *p* values involve comparison with FAB M5. *p < 0.05, **p 0.01, ***p 0.001. Statistical significance was determined by a two-sided unpaired Student`s *t* test. The variance was similar between the groups.

To further assess the effect of RSK inhibition on anti-apoptotic proteins, we performed an intracellular staining for BCL-2 family proteins (i.e. MCL1, BCL2 and BCL-xL) in HL-60 cells following treatment with venetoclax and azacitidine with and without the two RPS6KA1 inhibitors BI-D1870 and SL0101. Following intracellular staining with fluorophore-tagged antibodies, expression of anti-apoptotic proteins was analyzed by flow cytometry and normalized relative to the expression in untreated cells. We observed that cells treated with BI-D1870 underwent a G2 arrest, which is a known effect of RSK inhibition in AML resulting in apoptosis as described for BI-D1870 [38]. Therefore, we analyzed the amount of BCL-2 family proteins in total cells alive and in cells in G1 phase. We observed that MCL-1 levels increased upon treatment with venetoclax-azacitidine and decreased upon RSK inhibition by either BI-D1870 or SL0101 (Fig. 5A, Supplementary Fig. S4E). Levels of BCL-2 and BCL-XL, however, remained rather stable (Fig. 5A, Supplementary Fig. S4E).

### RPS6KA1 inhibition specifically targets monocytic subpopulations

AML is a genetically heterogeneous disease in which various subclones with distinct mutations may coexist at the same time. Therapy resistance can arise from certain subclones that are not eliminated by first line treatment and outgrow under therapeutic pressure. From CFU assays performed under different treatment conditions, parental OCI-AML2 cells were isolated, stained with fluorophore-tagged antibodies for detection of myeloid differentiation markers such as CD11b, CD64, CD34, CD117 and GPR56, and analyzed by flow cytometry. Basically, two populations were detected in untreated OCI-AML2 cells, one CD45 high and one with CD45 dim expression. Upon treatment with BI-D18-70, the CD45-dim population was partially eradicated and completely gone upon treatment with the triple combination (Supplementary Fig. S5A). Most surface markers analyzed were unchanged upon different therapies except for the CD11b + CD64+ subset (Supplementary Fig. S5B). Within the CD45-high population, the CD11b- and CD64- negative population was partially positive for CD117 but negative for CD34 and GPR56 (Supplementary Fig. S5C and S5D).

Interestingly, in untreated colonies, a CD64^+^ subpopulation was present, indicative for monocytic differentiation. Upon venetoclax/azacitidine treatment this population was only slightly reduced (Fig. 5B, C). A recent study of Pei et al. demonstrated that monocytic differentiation is associated with venetoclax/azacitidine resistance and monocytic subpopulations are enriched in venetoclax/azacitidine refractory patient samples [28]. Further, a preexisting monocytic clone was prone to confer upfront resistance to venetoclax/azacitidine or outgrow upon therapy and therefore caused relapse [28]. Of note, colonies grown under BI-D1870 exposure comprised a significantly smaller fraction of CD64- expressing cells compared to untreated and venetoclax/azacitidine- treated samples, which was even decreased by venetoclax/azacitidine/BI-D1870 triple treatment (Fig. 5C). In line, mRNA expression levels of *RPS6KA1* were particularly high in AML patients with the monocytic M5 subtype (Fig. 5D).

We further compared the differentiation state of parental and resistant OCI-AML2 cells. Of note, the monocytic marker CD14 was expressed on a higher level in the resistant cells (Supplementary Fig. S5E), underlining that outgrowth of monocytic subpopulations may enhance resistance against venetoclax.

### RPS6KA1 is upregulated in AML with acquired resistance to venetoclax/azacitidine

**Fig. 6:**
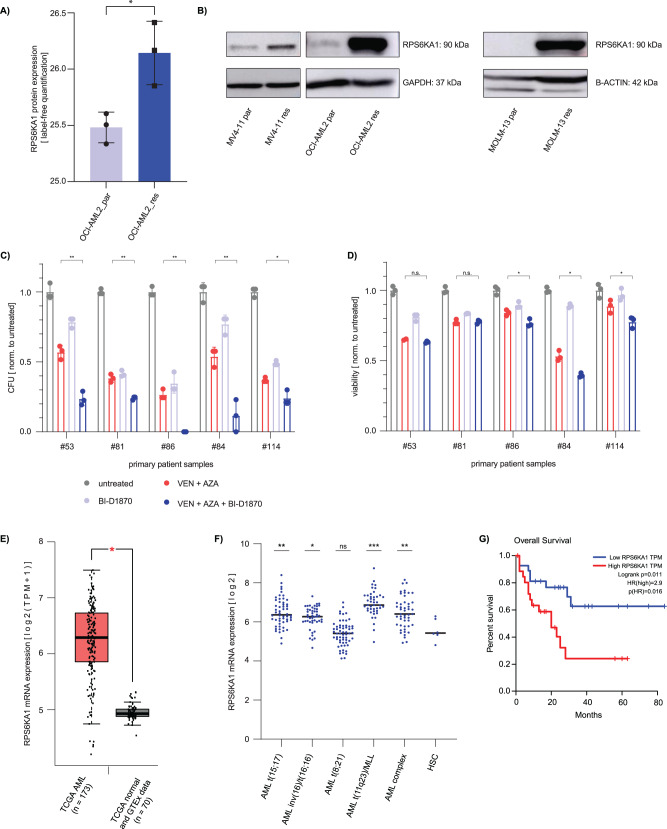
Increased RPS6KA1 expression is associated with resistance to venetoclax/azacitidine and a worse overall survival whereas RPS6KA1 inhibition enhances the effect of venetoclax/azacitidine in primary AML samples. **A** Label-free mass spectrometric analysis of RPS6KA1 protein expression in parental (wt) and resistant (res) OCI-AML2 cells. Depicted are means ± SD from three technical replicates. *p < 0.05, **p 0.01, ***p 0.001. Statistical significance was determined by a two-sided unpaired Student`s *t* test. The variance was similar between the groups. **B** RPS6KA1 western blot in three different cell lines with acquired resistance to venetoclax/azacitidine. GAPDH and B-ACTIN expression, respectively, served as loading control. Blots are representative for three independent experiments. **C** Colony forming unit (CFU) assay from primary AML patient samples. 4000 cells were seeded in methylcellulose and treated with venetoclax/azacitidine, BI-D1870 or the combination of all three drugs. Colonies with at least 50 cells were quantified 8 days after seeding and normalized to untreated samples. Depicted are means ± SD from three technical replicates. *p < 0.05, **p 0.01, ***p 0.001. Statistical significance was determined by a two-sided unpaired Student`s *t* test. The variance was similar between the groups. **D** Viability assay from primary AML patient samples. Two samples from newly diagnosed AML patients (#84, #114) who responded to venetoclax/azacitidine treatment and three samples from relapsed AML patients (#53, #81, #86) after venetoclax/azacitidine treatment were treated with venetoclax/azacitidine, BI-D1870 or the combination of all three drugs. Cell viability was assessed upon drug exposure for 48 h and normalized to untreated control. Depicted are means ± SD from three technical replicates. *p < 0.05, **p 0.01, ***p 0.001. Statistical significance was determined by a two-sided unpaired Student`s t test. The variance was similar between the groups. **E** *RPS6KA1* mRNA expression levels in AML and healthy bone marrow. *RPS6KA1* mRNA expression data are derived from the TCGA-AML dataset and the TCGA and GTEx data set of normal tissue [39] *p < 0.05. Statistical significance was determined by a two-sided unpaired Student`s *t* test. The variance was similar between the groups. **F** *RPS6KA1* mRNA expression levels in AML with different cytogenetical aberrations [40]. Compared to healthy hematopoietic stem cells (HSC), *RPS6KA1* expression was increased in AML with the mutations t(15;17), inv(16)/t(16;16), t(11q23)/MLL and in AML with complex karyotype. *p < 0.05, **p 0.01, ***p 0.001. Statistical significance was determined by a two-sided unpaired Student`s *t* test. The variance was similar between the groups. **G** Kaplan-Meier-Plot showing overall survival. High *RPS6KA1* mRNA expression in AML patients correlates with a shorter overall survival compared to patients with low *RPS6KA1* mRNA expression. For survival analysis the TCGA-AML dataset was used and the quartile with the highest *RPS6KA1* expression (*n* = 27) was compared to the quartile with the lowest *RPS6KA1* expression (*n* = 27) [39]. Log-rank test.

Next, the proteome of parental OCI-AML2 cells and their resistant counterparts was analyzed by mass spectrometry. RPS6KA1 protein expression levels were significantly higher in OCI-AML2 cells with acquired venetoclax/azacitidine resistance compared to parental cells (Fig. 6A). This finding was confirmed by western blot in parental and resistant OCI-AML2 cells and further validated in parental and resistant counterparts of the AML cell lines MV4-11 and MOLM-13 (Fig. 6B). Data acquired by mass spectrometry were further analyzed by GSEA.

### AML patients respond to venetoclax/azacitidine plus RPS6KA1 inhibition in vitro

To assess if combination of RPS6KA1 inhibition with venetoclax/azacitidine affects AML patient samples in vitro, we performed viability and colony forming unit (CFU) assays using primary samples. We chose two samples from patients at the time of first diagnosis of AML and three samples from patients with refractory/relapsed AML to venetoclax and azacitidine at the time point of relapse. Patient characteristics are summarized in Supplementary Table S1.

Colony formation assays showed reduction of colony formation capacity by addition of the RPS6KA1 inhibitor in all patient samples (Fig. 6C, Supplementary Table S7). In the patient samples with newly diagnosed AML, we observed a significantly reduced viability with the triple combination (Fig. 6D). Of the three relapse samples, the synergistic activity of venetoclax/azacitidine and RPS6KA1 inhibition could only be confirmed in one sample in the dose response assay (patient #86, Fig. 6D).

### RPS6KA1 is highly expressed in AML patient samples and is associated with a worse prognosis

We further analyzed mRNA expression of *RPS6KA1* in healthy bone marrow and different AML subtypes and the association between *RPS6KA1* expression and patient outcomes. First, we performed a differential expression analysis of RNA sequencing data comparing AML samples and healthy tissue using Gene Expression Profiling Interactive Analysis (GEPIA) [39]. This analysis showed that *RPS6KA1* mRNA expression was higher in AML compared to healthy hematopoietic cells (Fig. 6E) [39]. Similar results were obtained by a comparative analysis using the database BloodSpot and AML samples harboring different cytogenetic aberrations [40]. *RPS6KA1* was significantly upregulated in AML with t(15;17), inv(16)/t(16;16), t(11q23)/MLL and AML with complex karyotype compared to healthy hematopoietic stem cells (Fig. 6F). Further, survival analysis with GEPIA revealed that patients with a high mRNA expression of *RPS6KA1* had worse overall survival compared to patients with low *RPS6KA1* mRNA expression (Fig. 6G) [39].

## Discussion

The combination of the BCL-2 inhibitor venetoclax and the hypomethylating drug azacitidine constitutes the standard of care for patients with newly diagnosed AML who are at least 75 years old or who have preexisting comorbidities precluding them from intensive chemotherapy [9]. The combination is effective, yielding composite response rates of 67% and a median survival of 17.5 months [2]. However, upfront resistance as well as relapse after initial response remain unsolved problems, and additional combination partners for venetoclax/azacitidine are needed to overcome resistance [10]. We performed a CRISPR/Cas9 library screen to identify genetic alterations which may affect sensitivity to treatment and identified the ribosomal protein kinase RPS6KA1 as mediator of venetoclax/azacitidine resistance. Resistance towards venetoclax monotherapy but not to combination therapy was previously investigated earlier using similar genome-wide approaches. Here, knockout of *TP53*, *BAX*, and *PMAIP1* were found to induce venetoclax resistance by inducing alterations in energy metabolism and the apoptosis cascade [11, 16]. In addition, CLPB was identified as a potential drug target for venetoclax combination therapies, as its overexpression caused venetoclax resistance and its inhibition led to loss of mitochondrial ultrastructure and apoptosis induction [11]. RPS6KA1 was not identified in these venetoclax monotherapy CRISPR/Cas9 library screens (Supplementary Fig. S2A-B) indicating that resistance mechanisms for venetoclax mono and venetoclax/azacitidine may differ.

We validated RPS6KA1 inhibition by BI-D1870 as potential combination partner for venetoclax/azacitidine and demonstrated that inhibition of proliferation and colony growth of AML cells was strongly enhanced under triple therapy. These findings could be confirmed in primary patient samples. Moreover, we showed that RPS6KA1 inhibition was able to eliminate subclones with monocytic differentiation that endured venetoclax/azacitidine treatment.

Resistance to venetoclax is often associated with the upregulation of MCL-1 [36, 37]. We found the inhibition of RPS6KA1 to increase activity of GSK3 resulting in increased phosphorylation of MCL-1 at serine 159 with subsequent degradation of MCL-1 (Fig. 4). The downregulation of MCL-1 has been shown to be a promising strategy for combination therapy with venetoclax in various approaches [35, 37, 41, 42]. However, direct inhibition of MCL-1 is linked with high toxicity in vivo [43] so attempts to indirectly inhibit MCL-1 appear to be most promising for combination therapy with venetoclax. As RPS6KA1 is a downstream target in the Ras/Raf/MAPK signaling pathway, it might be suspected to have less toxic effects in vivo than upstream inhibition of this pathway. Therefore, the approach might be effective as a triple therapy approach. Our results suggest, however, that a dual inhibition of BCL-2 and RPS6KA1 might be highly efficient as well and merits further investigation.

It appears likely that decrease in viability of AML and targeting of monocytic subclones is linked to further functions of RPS6KA1. Its kinase activity mediates activation of transcription factors such as CREB1 or inactivates GSK3B, but also invokes translation via post-translational modification of RPS6 and EIF4B, leading to pre-initiation complex assembly and guiding the 40 S ribosomal subunit to the mRNA [44]. Via mTOR signaling and repression of the pro-apoptotic proteins BAD and DAPK1, RPS6KA1 mediates cellular proliferation, survival, and differentiation [29, 38, 44]. Cell cycle progression is affected by RPS6KA1 mediated phosphorylation of the CDK inhibitor CDKN1B, preventing nuclear translocation [45]. Beyond, apoptosis induction is inhibited by RPS6KA1 via modification of proapoptotic factors such as BAD and BIM [45, 46]. In breast as well as in lung cancer, it was shown that high phosphorylation levels of EPHA2 induced by RPS6KA1 enhance cellular migration and tissue invasion capacity [44]. There is already evidence that overexpression of *RPS6KA1*—independently or in the context of FLT3-ITD mutation—is associated with worse prognosis, poor survival rates, and chemoresistance of AML patients [47]. Inhibition of RPS6KA1 either directly or by targeting upstream signaling pathways is expected to sensitize AML cells to BCL-2 inhibition [47]. The inhibitor we used for our validation experiments, BI-D1870, is potent and highly specific for RSK isoforms in vitro and in vivo. In a kinase selectivity screen, concentrations resulting in 98% inhibition of the four RSK isoforms were shown to not affect 50 other tested protein kinases [48]. BI-D1870 was classified as antimitotic drug inducing metaphase arrest and therefore acts synergistically with vincristine, for example [38].

In summary, the findings of this study suggest RPS6KA1 as novel therapeutic target in AML. Inhibition of this ribosomal protein kinase in addition to venetoclax/azacitidine may be a promising therapeutic approach for upfront resistant or relapsed AML patients. A limitation of our study is the lack of in vivo experience with RPS6KA1 inhibitors as therapeutic agents. Currently, it is not known if BI-D1870 or related compounds can be administered at an appropriate dosage and treatment period to achieve similar on-target effects without unacceptable toxicity and side effects. Especially, interdependences with simultaneous venetoclax/azacitidine treatment in humans are so far unknown. Further, observation time in our in vitro studies was relatively short, thus not yet allowing translation into long-term treatment, with possible recurrent resistance and toxicity, and in vivo studies such as xenograft transplantation models need to be performed to validate the effects of RPS6KA1 inhibition in vivo. Nevertheless, findings from this study suggest that patients with a high RPS6KA1 expression prior to therapy might be classified as high-risk patients for developing relapse or a resistant disease under venetoclax/azacitidine treatment. Those patients could be offered a more intensive or adapted therapy and might represent potential candidates for a triple therapy with venetoclax/azacitidine and a RSK inhibitor, such as PMD-026 which is currently evaluated in a phase I clinical trial in triple negative breast cancer patients [49].

## Supplementary information

Supplemental Figures

Supplementary Tables S2-S6

## Data Availability

Primer sequences for PCR amplification of genomic DNA, results of next generation sequencing and analyses with the MaGeCK algorithm are included in this article and its supplementary information files, further datasets are available from the corresponding author on reasonable request.
