# Supplementary material for: The ribosomal protein S6 kinase alpha-1 (RPS6KA1) induces resistance to venetoclax/azacitidine in acute myeloid leukemia
Source: Leukemia. 2023 Jul 6;37(8):1611–25. doi: 10.1038/s41375-023-01951-8 (PMC10400424; doi:10.1038/s41375-023-01951-8)
Supplement: Supplementary file 1 — Supplemental Figures [file 41375_2023_1951_MOESM1_ESM.pdf]

Supplementary figures

Supplementary Figure S1. Data analysis of the CRSIPR/Cas9 library screen.

A Sequencing results of the OCI-AML2 CRISPR/Cas9 screen shown as the proportional read counts for each sgRNA included in the screen from 1.3 x 10^7 reads in total. Shown are all technical replicates in blue and the original TKOv3 library as black line. More than 61 000 sgRNAs have a coverage of more than 100 reads.

B Waterfall plot showing LFC values for all sgRNAs between treatment and control samples on day 14 after start of treatment.

C Volcano plot (left) for median LFC values between day 14 and day 0 and the corresponding p-value for all sgRNAs per gene in the DMSO treated samples (control). Selected sgRNA-genes are shown in a bar plot (right) with median LFC values for all targeting sgRNAs. Highlighted are essential genes in red, non-targeting genes in orange and positively enriched sgRNA-genes in blue.

D sgRNA-genes ranked by negative (left) and positive (right) MaGeCK score and p-value for the comparison of treated and untreated samples on day 14. RPS6KA1 is among the top negatively ranked sgRNA-genes. Highlighted are non-targeting and positively enriched sgRNA-genes in orange and blue, respectively.

Supplementary Figure S2. Functional assays in parental AML cell lines validate BI-D1870 as novel drug combination partner for venetoclax/azacitidine.

A MaGeCK ranking for negative enrichment of selected genes in CRISPR/Cas9 screens investigating venetoclax resistance. TP53, BAX and PMAIP1 were constantly identified among the top enriched genes (high negative rank) in different venetoclax mono CRISPR/Cas9 screens as well as in the venetoclax/azacitidine CRISPR/Cas9 screen presented in this study. Previously, CLPB (1) and DAP3 (2) were further validated as genes mediating resistance to venetoclax therapy, while TP53 was investigated as mediator of venetoclax response (3).

B Median MaGeCK rank for negative enrichment of sgRNAs targeting TP53, BAX, PMAIP1 and RPS6KA1 in screens investigating venetoclax mono resistance versus the venetoclax/azacitidine screen presented in this study. Data indicate

that RPS6KA1 could specifically mediate venetoclax/azacitidine resistance but not venetoclax mono resistance.

C Negative normalized enrichment scores of three gene sets related to biological functions of RPS6KA1 as analysed by GSEA.

D Venetoclax/azacitidine response in dependence of BI-D1870 treatment. IC50 of venetoclax/azacitidine (fixed 1:4 combination was used) was calculated in several cell lines under treatment with different concentrations of the RPS6KA1 inhibitor BI-D1870.

E Parental (wt) Kasumi-1 and MV4-11 cells were treated with a premixed combination of venetoclax/azacitidine in serial dilutions and different concentrations of the RPS6KA1 inhibitor BI-D1870. Cell viability was assessed upon drug treatments for 72 hours and normalized to DMSO control. Depicted are means ± SD from three technical replicates. Data are representative for three independent experiments.

F Synergism was calculated for different drug combinations of venetoclax/azacitidine with BI-D1870 in parental (wt) Kasumi-1 and MV4-11 cells. The synergy score was computed according to the Bliss Independence Model and depicted as a heatmap. Positive and negative Bliss scores are indicative for synergism and antagonism, respectively, while neutral Bliss scores are often confounded by strong single drug effects.

Supplementary Figure S3. BI-D1870 increases apoptosis induction when combined with venetoclax or venetoclax/azacitidine.

A-D OCI-AML2 (A, C) and MOLM-13 (B, D) cells were treated for 48 (A, B) or 72 hours (C, D) with 50 nM venetoclax, 200 or 500 nM azacitidine or the combination of both with or without 2 µM BI-D1870. Apoptosis was measured by staining cells for Annexin V and PI and analysing cells by flow cytometry. Images are exemplary for technical triplicates and two independent experiments.

E-F OCI-AML2 and MOLM-13 cells were treated with 50 nM venetoclax or 50 nM venetoclax and 200 nM azacitidine with or without 2 µM BI-D1870 for 48 hours (E) or with 500 nM azacitidine or 100 nM venetoclax and 500 nM azacitidine with or without 2 µM BI-D1870 for 72 hours (F). Fraction of apoptotic cells was analysed by Annexin/PI staining with flow cytometry and is given as mean ± SD

from three technical replicates. Statistical significance was determined by a two-sided unpaired Student`s t-test. ** $p < 0.005$. *** $p < 0.0005$. **** $p < 0.00005$.

Supplementary Figure S4. BI-D1870 influences BCL-2 family proteins when combined with venetoclax/azacitidine

A Parental OCI-AML2 cells were treated with increasing doses of venetoclax/azacitidine in a 1:4 ratio. 1 µM BI-D1870 or 10 or 50 µM of the RSK inhibitor SL0101 were added. Viability is shown as mean ± SD from three technical replicates. Data is representative for two independent experiments.

B Parental OCI-AML2 cells were rendered resistant towards venetoclax/azacitidine treatment by weekly treatment with increasing doses of the drugs. Resistance was confirmed by an increased IC50 concentration. It was determined by treating parental and resistant cells with different concentrations of venetoclax and azacitidine for 48 hours. Viability is shown as mean ± SD from three technical replicates. Data is representative for two independent experiments.

C-D OCI-AML2 (C) and HL-60 (D) cells were treated with given dose of venetoclax, azacitidine and/or BI-D1870 for 16 hours. Protein expression of pMCL-1 T163 was analyzed by western blotting. Quantification was performed using ImageJ. Data are representative for two independent experiments.

E HL-60 cells were treated with venetoclax (50 nM) and azacitidine (100 nM) with or without 10 µM BI-D1870 or 50 µM SL0101 for 16 hours. Levels of BCL-2, BCL-XL and MCL-1 were determined by intracellular staining and analysis via flow cytometry. Analysis was carried out for cells in G1 state. Data is shown as mean ± SD from three technical replicates.

Supplementary Figure S5. Flow cytometric analysis of myeloid subpopulations.

A-D Flow cytometric analysis of cell surface markers. Parental (wt) OCI-AML2 cells were isolated after CFU assays under different treatment conditions, stained with a panel of myeloid differentiation markers (CD11b, CD64, CD117, GPR56 and CD34) and analyzed by flow cytometry. FACS plots are representative for one technical replicate out of three showing expression levels of the various differentiation markers. CD45 in different treatment conditions is shown in (A). Expression of CD11b and CD64 in the CD45 high population are depicted in

(B). The nonCD11bCD64 population was further analysed for CD34 and CD117 (C) as well as for CD34 and GPR56 (D).

E Analysis of CD14 cell surface marker by flow cytometry. The fraction of CD14+/7-AAD- cells is higher in resistant (res) OCI-AML2 cells compared to parental (par) OCI-AML2 cells indicative for a more dominant subpopulation with monocytic differentiation.

Supplementary Tables

Supplementary Table 1: Characteristics of primary AML samples that were analyzed.

Supplementary Table 2: Primer sequences used for two-step PCR in CRIPR/Cas9 screen.

Supplementary Table 3: sgRNA-ranking comparing treatment and control samples based on p-values

Supplementary Table 4: MaGeCK analysis showing negative and positive enrichment in control samples day 14 versus day 0

Supplementary Table 5: MaGeCK analysis showing negative and positive enrichment in treated samples day 14 versus day 0

Supplementary Table 6: MaGeCK analysis showing negative and positive enrichment in treated versus untreated samples at day 14

Supplementary Table 7: Absolute numbers of colonies counted in CFU assays conducted with primary patient samples.

References

1. Chen X, Glytsou C, Zhou H, Narang S, Reyna DE, Lopez A, et al. Targeting Mitochondrial Structure Sensitizes Acute Myeloid Leukemia to Venetoclax Treatment. Cancer Discov. 2019;9(7):890-909.
2. Sharon D, Cathelin S, Mirali S, Di Trani JM, Yanofsky DJ, Keon KA, et al. Inhibition of mitochondrial translation overcomes venetoclax resistance in AML through activation of the integrated stress response. Sci Transl Med. 2019;11(516).
3. Nechiporuk T, Kurtz SE, Nikolova O, Liu T, Jones CL, D'Alessandro A, et al. The TP53 Apoptotic Network Is a Primary Mediator of Resistance to BCL2 Inhibition in AML Cells. Cancer Discov. 2019;9(7):910-25.

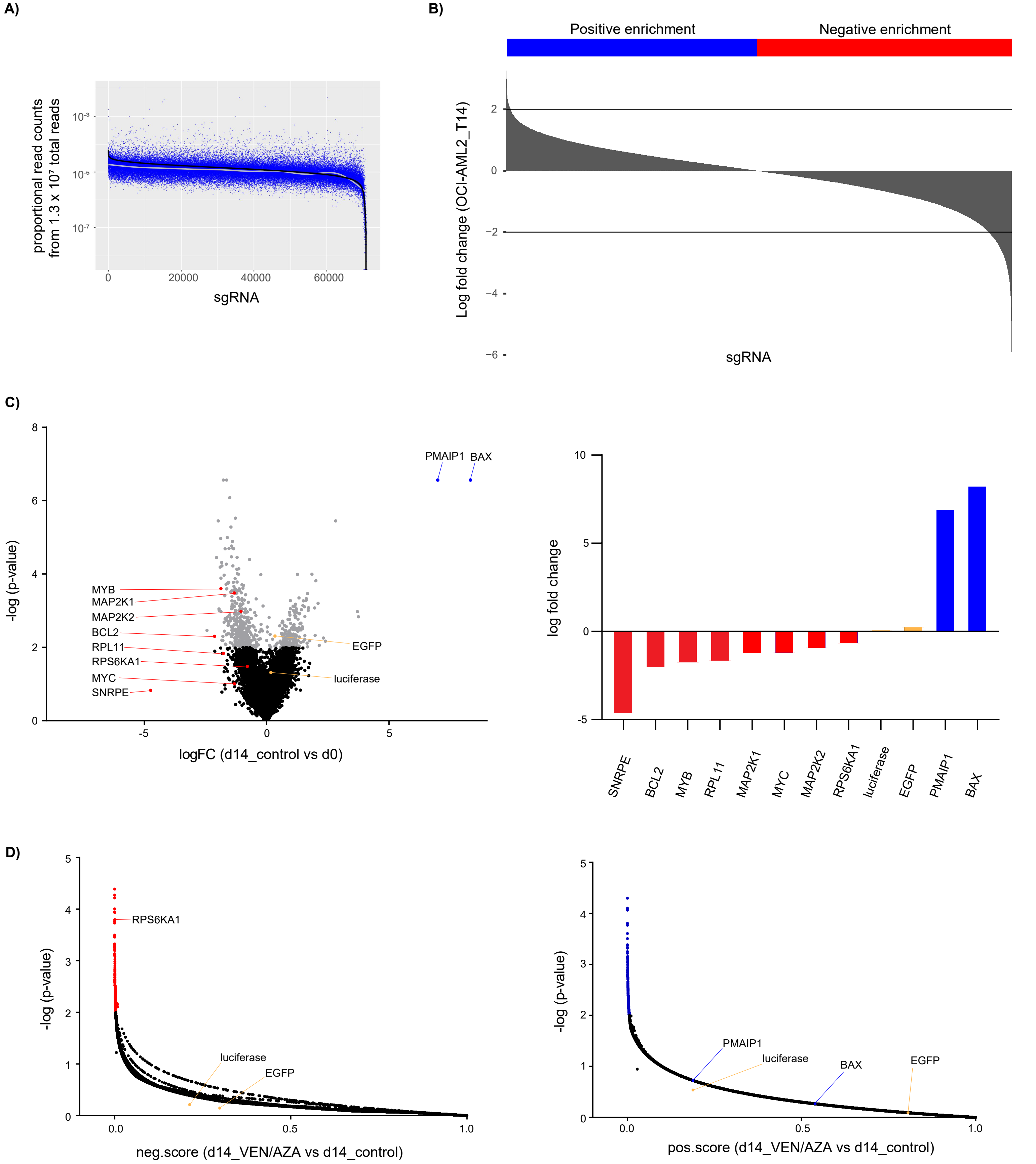

Supplementary Figure S1

A)

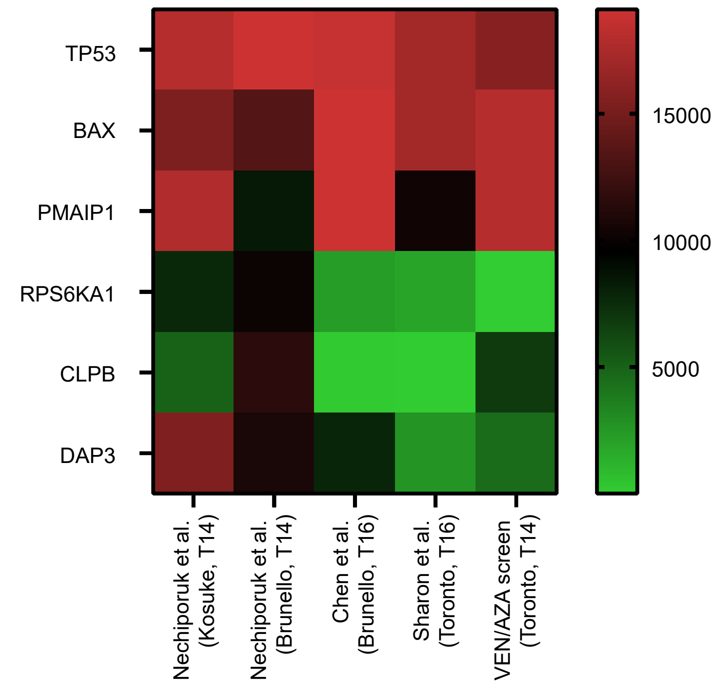

B)

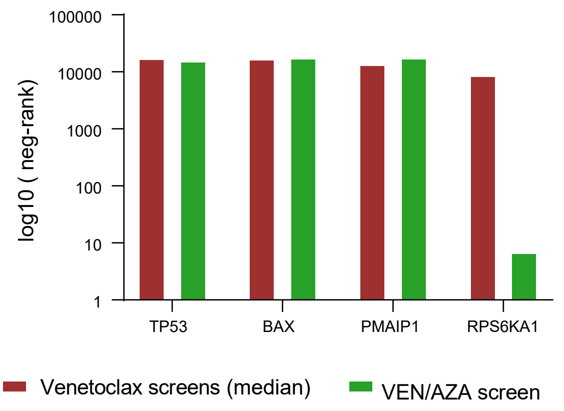

C)

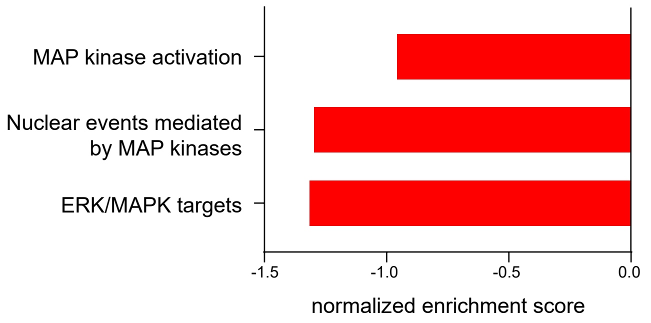

D) IC_{50} [x 2.5 nM Venetoclax / 10 nM Azacitidine]

	0 nM BI-D1870	1 µM BI-D1870	2 µM BI-D1870
OCI-AML2_wt	42.44	23.67	16.99
OCI-AML2_res	776.9	558.2	398.7
MOLM-13	10.20	5.954	2.046
HL-60	3087	1207	116.4
MV4-11	112.7	53.67	59.06
Kasumi-1	1.158	0.09017	0.00001803

E)

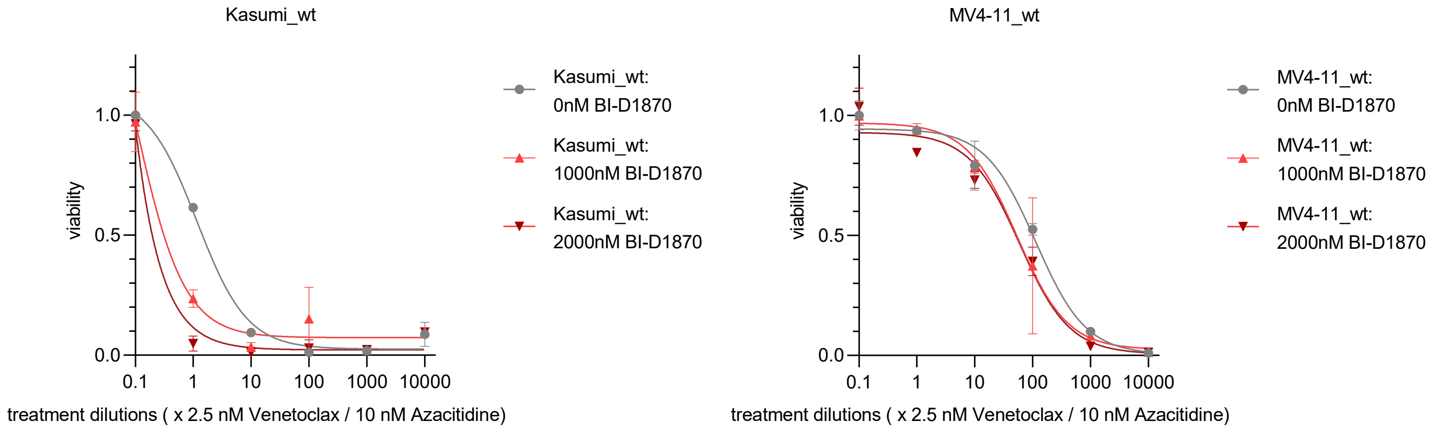

F)

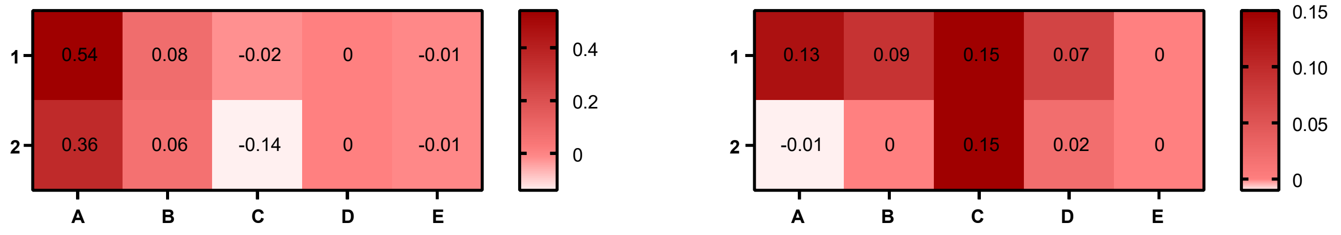

Supplementary Figure S2

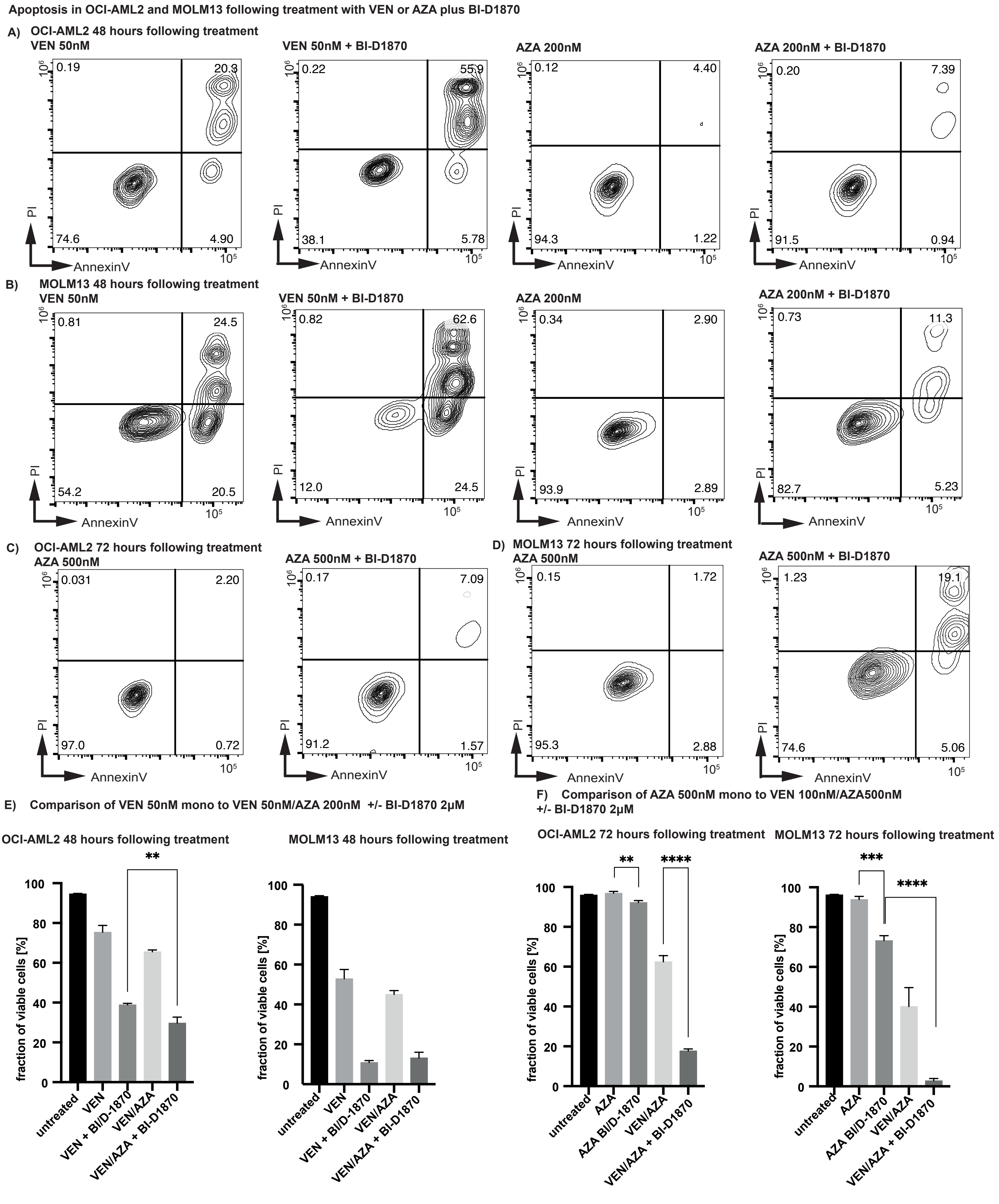

Supplementary Figure S3

A)

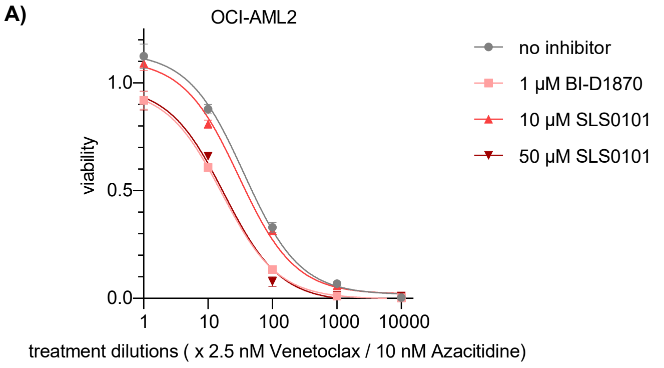

B)

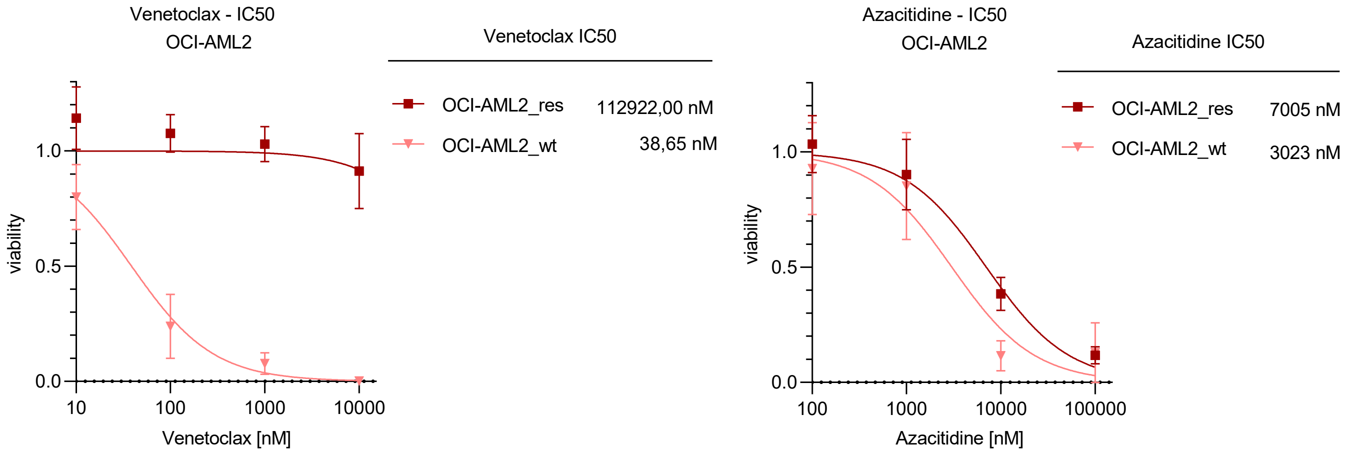

C)

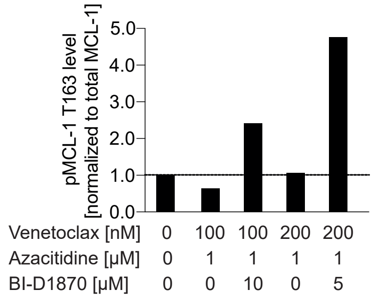

D)

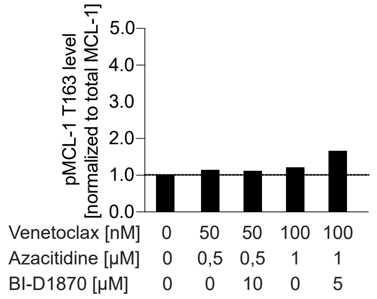

E)

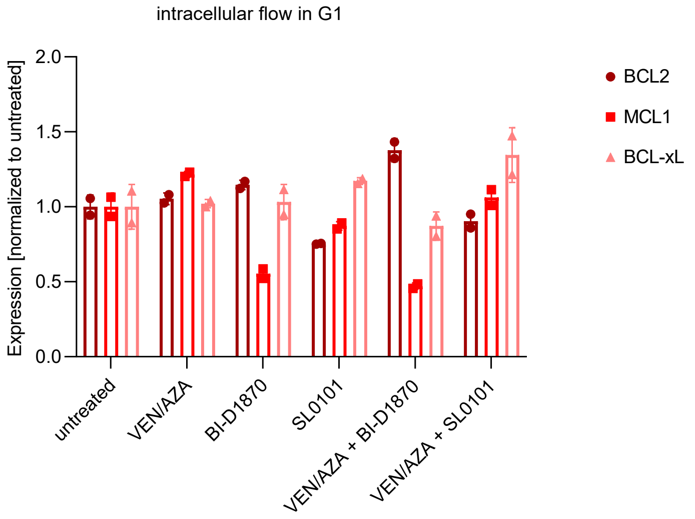

Supplementary Figure S4

Panel analysis of cell surface markers post CFU assay of OCI-AML2

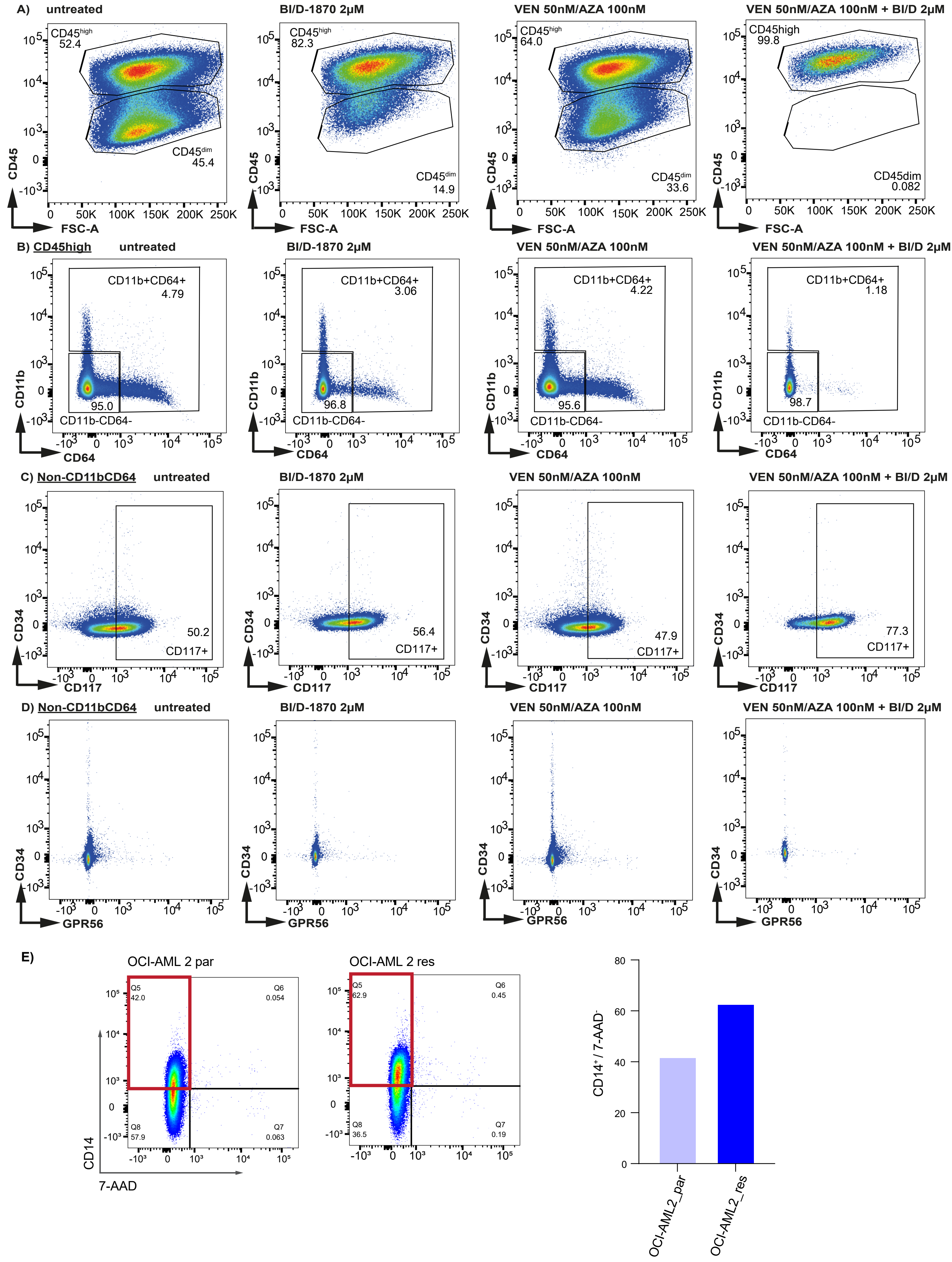

Supplementary Figure S5

Supplementary Table S1

Sampling timepoint	ID	Firstline Treatment	Firstline Response	Salvage Treatment	Salvage Response	EFS (days)	Age (years)	Female	Karyotype	PB Leukocyte counts (/nl)	BM blasts (%)	Mutations (Myeloid panel)
sample at relapse	53	5-AZA/VEN	CR/MLFS	1st Vyxeos, 2nd High-dose cytarabine+mitoxantrone	PD	39	45	yes	46,XX	11.5	70	TET2, DNMT3A, KRAS, WT1
first diagnosis	84	5-AZA/VEN + allo SCT --> relapse day 100	CR/MLFS	FLAG-IDA	CR/MLFS	189	62	no	46,XY,del5q(q31q35)	87	82	NRAS
sample at relapse post 5-AZA/VEN	86	DA+Midostaurin	refractory	5-AZA/VEN	CR/MLFS	90	59	yes	46, XX	42.6	60	NPM1, FLT3-ITD (Ratio 0,95), DMT3A
first diagnosis	114	6x 5-AZA/VEN --> CR	CR/MLFS	NA	NA	210	72	yes	46, XX	56	71	NPM1, TET2
sample at relapse	81	14 x 5-AZA/VEN--> relapse	CR/MLFS	NA	NA	730	73	no	46,XY	21	40	RUNX1, SF3B1

Supplementary Table S7

sample	treatment	colonies_rep1	colonies_rep2	colonies_rep3
#53	untreated	30	33	30
#53	BI-D1870	23	25	25
#53	VEN/AZA	19	18	16
#53	VEN/AZA + BI-D1870	6	9	7
#81	untreated	144	139	139
#81	BI-D1870	62	56	57
#81	VEN/AZA	54	50	58
#81	VEN/AZA + BI-D1870	35	32	35
#84	untreated	8	9	9
#84	BI-D1870	7	6	7
#84	VEN/AZA	5	4	5
#84	VEN/AZA + BI-D1870	2	1	0
#86	untreated	16	17	16
#86	BI-D1870	5	7	5
#86	VEN/AZA	5	4	4
#86	VEN/AZA + BI-D1870	0	0	0
#114	untreated	34	32	34
#114	BI-D1870	17	16	16
#114	VEN/AZA	12	12	13
#114	VEN/AZA + BI-D1870	7	10	7
